# Effectiveness and safety of bimekizumab for the treatment of plaque psoriasis: a real-life multicenter study—IL PSO (Italian landscape psoriasis)

**DOI:** 10.3389/fmed.2023.1243843

**Published:** 2023-08-08

**Authors:** Luigi Gargiulo, Alessandra Narcisi, Luciano Ibba, Anna Balato, Luca Bianchi, Pina Brianti, Dario Buononato, Martina Burlando, Giacomo Caldarola, Anna Campanati, Elena Campione, Carlo G. Carrera, Andrea Carugno, Antonio Cristaudo, Francesco Cusano, Paolo Dapavo, Annunziata Dattola, Clara De Simone, Francesca M. Gaiani, Paolo Gisondi, Alessandro Giunta, Francesco Loconsole, Vincenzo Maione, Edoardo Mortato, Angelo V. Marzano, Martina Maurelli, Matteo Megna, Santo R. Mercuri, Annamaria Offidani, Diego Orsini, Aurora Parodi, Giovanni Pellacani, Luca Potestio, Pietro Quaglino, Antonio G. Richetta, Francesca Romano, Paolo Sena, Marina Venturini, Piergiorgio Malagoli, Antonio Costanzo

**Affiliations:** ^1^Dermatology Unit, IRCCS Humanitas Research Hospital, Milan, Italy; ^2^Department of Biomedical Sciences, Humanitas University, Milan, Italy; ^3^Dermatology Unit, University of Campania L. Vanvitelli, Naples, Italy; ^4^Dermatology, Department of Systems Medicine, University of Rome Tor Vergata, Rome, Italy; ^5^Unit of Dermatology and Cosmetology, Vita-Salute San Raffaele University and Institute for Research and Care, Milan, Italy; ^6^Section of Dermatology, Department of Health Sciences (DISSAL), IRCCS San Martino University Hospital, Genoa, Italy; ^7^Section of Dermatology, Department of Translational Medicine and Surgery, Catholic University of the Sacred Heart, Rome, Italy; ^8^Dermatology Unit, Agostino Gemelli University Hospital, Rome, Italy; ^9^Department of Clinical and Molecular Sciences—Dermatological Clinic, Università Politecnica delle Marche, Ancona, Italy; ^10^Dermatology Unit, Fondazione IRCCS Ca’ Granda Ospedale Maggiore Policlinico, Milan, Italy; ^11^Dermatology Unit, ASST Papa Giovanni XXIII Hospital, Bergamo, Italy; ^12^PhD Program in Molecular and Translational Medicine (DIMET), University of Milan-Bicocca, Milan, Italy; ^13^Clinical Dermatology Unit, San Gallicano Dermatological Institute IRCCS, Rome, Italy; ^14^Department of Dermatology, Gaetano Rummo Hospital, Benevento, Italy; ^15^Department of Biomedical Science and Human Oncology, Second Dermatologic Clinic, University of Turin, Turin, Italy; ^16^Dermatology Clinic, Department of Clinical Internal, Anesthesiological and Cardiovascular Sciences, Sapienza University, Rome, Italy; ^17^Department of Dermatology, Dermatology Unit Azienda Ospedaliera San Donato Milanese, Milan, Italy; ^18^Department of Medicine, Section of Dermatology and Venereology, University of Verona, Verona, Italy; ^19^Department of Dermatology, University of Bari, Bari, Italy; ^20^Department of Dermatology, ASST Spedali Civili Hospital, Brescia, Italy; ^21^Department of Pathophysiology and Transplantation, Università degli Studi di Milano, Milan, Italy; ^22^Section of Dermatology, Department of Clinical Medicine and Surgery, University of Naples Federico II, Naples, Italy; ^23^Department of Precision Medicine, Dermatology Unit, Università degli Studi della Campania L. Vanvitelli, Naples, Italy

**Keywords:** biologics, bimekizumab, psoriasis, psoriasis treatment, real-life

## Abstract

**Introduction:**

Bimekizumab is a monoclonal antibody that targets Interleukin-17 A and F, approved for the treatment of moderate-to-severe plaque psoriasis. While bimekizumab has been evaluated in several phase-III clinical trials, real-world evidence is still very limited.

**Method:**

This multicenter retrospective study included patients affected by plaque psoriasis treated with bimekizumab from May 1, 2022 to April 30, 2023, at 19 Italian referral hospitals. Patients affected by moderate-to-severe plaque psoriasis eligible for systemic treatments were included. The effectiveness of bimekizumab was evaluated in terms of reduction in psoriasis area and severity index (PASI) compared with baseline at weeks 4 and 16. The main outcomes were the percentages of patients achieving an improvement of at least 75% (PASI75), 90% (PASI90) and 100% (PASI100) in PASI score.

**Results:**

The study included 237 patients who received at least one injection of bimekizumab. One hundred and seventy-one patients and 114 reached four and 16 weeks of follow-up, respectively. Complete skin clearance was achieved by 43.3% and 75.4% of patients at weeks 4 and 16, respectively. At week 16, 86.8% of patients reported no impact on their quality of life. At week 16, there were no significant differences between bio-naïve and bio-experienced patients in terms of PASI75, PASI90 and PASI100. The most commonly reported adverse events (AEs) were oral candidiasis (10.1%). No severe AEs or AEs leading to discontinuation were observed throughout the study.

**Conclusion:**

Our experience supports the effectiveness and tolerability of bimekizumab in a real-world setting with similar results compared with phase-III clinical trials.

## 1. Introduction

Psoriasis is a chronic immune-mediated systemic disease primarily affecting the skin, presenting with erythematous scaly plaques and patches (1). The treatment of moderate-to-severe plaque psoriasis includes conventional systemic therapies and biological drugs (1). Several monoclonal antibodies were developed, targeting primarily two key cytokines in the pathogenesis of psoriasis: interleukin (IL)-23 and IL-17.1 IL-17A represents the target of two biological drugs (secukinumab and ixekizumab), while IL-17A receptor is antagonized by brodalumab (1). Recent research has focused on the role of IL-17F, which was found to have overlapping pro-inflammatory functions with IL-17A and to be even more expressed in psoriatic lesions (2).

Bimekizumab is a humanized monoclonal antibody that selectively targets both IL-17A and IL-17F. It has been approved for the treatment of moderate-to-severe plaque psoriasis after four phase-III clinical trials (be ready, be vivid, be sure, be radiant) demonstrated the superior efficacy of bimekizumab compared with placebo, ustekinumab, adalimumab and secukinumab (3–6). However, real-life data on bimekizumab are limited to case reports (7, 8).

To assess the effectiveness and safety of bimekizumab in a real-world setting, we conducted a 16 weeks retrospective multicenter study.

## 2. Method

Two hundred and thirty-seven patients from 19 Italian Dermatology Units received at least one injection of bimekizumab and were enrolled in this study. Patients’ characteristics were retrieved from the electronic records of each hospital.

Patients’ eligibility for bimekizumab treatment was evaluated according to Italian Guidelines (9). All patients were screened for viral hepatitis B and C, HIV and latent tuberculosis before starting the therapy. Bimekizumab was administrated according to the summary of product characteristics (two subcutaneous injections of 160 mg each, at weeks 0, 4, 8, 12, 16 followed by two injections every 8 weeks) in patients with inadequate response or contraindications to systemic treatments (10).

The procedures for this study adhered to clinical standards and did not necessitate approval from the institutional review board. All patients provided written consent for their anonymous data to be retrieved retrospectively. The research complied with the Helsinki Declaration of 1964 and its later amendments.

At each dermatological examination, psoriasis area and severity index (PASI) was assessed. The effectiveness of bimekizumab was evaluated at weeks 4 and 16 in terms of percentage reduction of 90 and 100% in PASI compared with baseline (PASI90 and PASI100, respectively). An additional endpoint was the proportion of patients achieving an absolute PASI of 2 or less at each visit. We conducted a sub-analysis to assess the effectiveness of bimekizumab in individuals who had previously undergone other biological treatments.

To evaluate the impact of bimekizumab on the quality of life of the patients, at each visit dermatology life quality index (DLQI) was recorded. The percentages of patients who achieved a DLQI of 0/1 (no impact on quality of life) were assessed at weeks 4 and 16.

At each visit, the occurrence of any adverse events (AEs) was investigated, including severe AEs and AEs leading to the discontinuation of bimekizumab.

Categorical data were reported as absolute frequencies and percentages, while continuous were presented as mean and standard deviation (SD).

Effectiveness and safety analyses were conducted on all patients who completed at least one follow-up visit (171 patients). The effectiveness of bimekizumab in terms of PASI ≤2, PASI90 and PASI100 was also evaluated according to previous exposure to other biologics. We used chi-square and exact Fisher’s tests to analyze categorical variables.

A significant *p*-value was defined as less than 0.05 in the study. STATA/SE 17.0 software was used to conduct the data analysis.

## 3. Results

Complete demographic characteristics and clinical features of all patients at baseline are reported in Table 1. The mean age of the patients was 48.86 years (SD 14.95), and 162 (68.4%) were males. They had a mean history of psoriasis of 15.04 years (SD 13.25) and a mean body mass index (BMI) of 26.93 kg/m^2^ (SD 5.21). More than half of patients (50.6%) had at least one cardio-metabolic comorbidity, including obesity, arterial hypertension, cardiovascular disease, type II diabetes mellitus and hypercholesterolemia. Twenty-five patients (10.6%) had a concomitant diagnosis of psoriatic arthritis. Four patients had positive tuberculosis (TB) Quantiferon test, with no clinical and radiological sign of active TB, before the start of biological drug. They were all evaluated by a pulmonologist and they received a 6 months prophylaxis before starting the treatment. Regarding the two patients with previous viral hepatitis, they were evaluated by the hepatologist and received the approval to start bimekizumab due to the absence of clinical and serological evidence of active disease. One hundred and thirty-four patients were naïve to biological therapies (56.5%).

**Table 1:** Demographic and clinical characteristics of our cohort at baseline.

Number of total patients	237
Male, No. (%)	162 (68.4)
Age, mean (SD), years	48.86 (14.95)
BMI, mean (SD), kg/m^2^	26.93 (5.21)
Obese (BMI ≥30), No. (%)	60 (25.3)
Disease duration, mean (SD), years	15.04 (13.25)
PsA, No. (%)	25 (10.6)
PASI at baseline, mean (SD)	16.96 (8.42)
DLQI at baseline, mean (SD)	15.49 (7.93)
DLQI ≥10, No. (%)	194 (81.9)
Difficult-to-treat areas, No. (%)	146 (61.6)
Cardiometabolic comorbidities, No. (%)	120 (50.6)
Hepatitis C, No. (%)	1 (0.4)
Hepatitis B, No. (%)	1(0.4)
TBC, No. (%)	4 (1.7)
Bio-experienced, No. (%)	103 (43.5)
Multi-failure, No. (%)	44 (18.6)
Anti-TNF-α, No. (%)	66 (27.9)
Adalimumab, No. (%)	45 (19.0)
Etanercept, No. (%)	15 (6.3)
Infliximab, No. (%)	6 (2.5)
Anti-IL-17, No. (%)	69 (29.1)
Secukinumab, No. (%)	31 (13.1)
Ixekizumab, No. (%)	22 (9.3)
Brodalumab, No. (%)	16 (6.8)
Anti-IL-23, No. (%)	32 (13.5)
Guselkumab, No. (%)	15 (6.3)
Tildrakizumab, No. (%)	3 (1.3)
Risankizumab, No. (%)	14 (5.9)
Ustekinumab, No. (%)	20 (8.4)
Apremilast, No. (%)	14 (5.9)

BMI, body mass index; PsA, psoriatic arthritis; PASI, psoriasis area and severity index; DLQI, dermatology life quality index.

**Figure 1:**
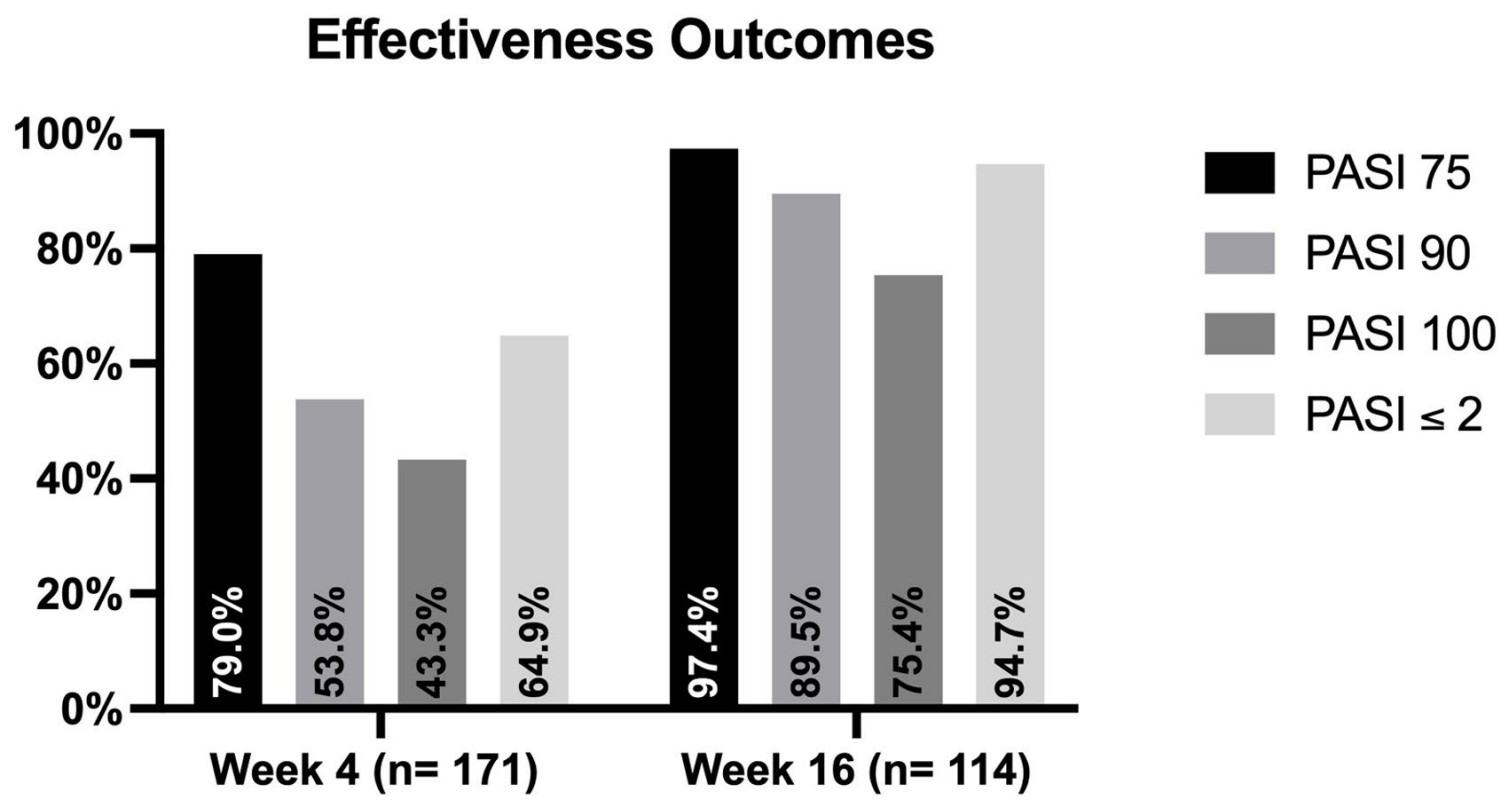
Proportion of patients who achieved PASI75, PASI90, PASI100 and PASI ≤2 at weeks 4 and 16. PASI, psoriasis area and severity index.

The mean PASI at baseline was 16.96 (SD 8.42). One hundred and forty-six patients (61.6%) had the involvement of at least one difficult-to-treat area (scalp/face, palms/soles, genital or nails). Severe impairment of the quality of life (with a DLQI ≥10) was reported by 194 patients (81.9%). Among the 171 patients who completed four weeks of follow-up, 92 (53.8%) and 74 (43.3%) achieved PASI90 and PASI100, respectively. An absolute PASI ≤2 was observed in 111 patients (64.9%). One hundred and fourteen patients completed 16 weeks of treatment. In this group, PASI ≤2, PASI90 and PASI100 were reached by 108 (94.7%), 102 (89.5%) and 86 patients (75.4%), respectively (Figure 1). The mean PASI decreased to 2.48 (SD 3.54) at week 4 and 0.52 (SD 1.15) at week 16.

The rapid improvement in PASI was also associated with a decrease in the mean DLQI throughout the study period, as it was 2.08 (SD 3.36) at week 4 and 0.48 at week 16 (SD 1.43). One hundred and three (60.2%) and 99 (86.8%) patients reported a DLQI of 0/1 after 4 and 16 weeks, respectively.

**Figure 2:**
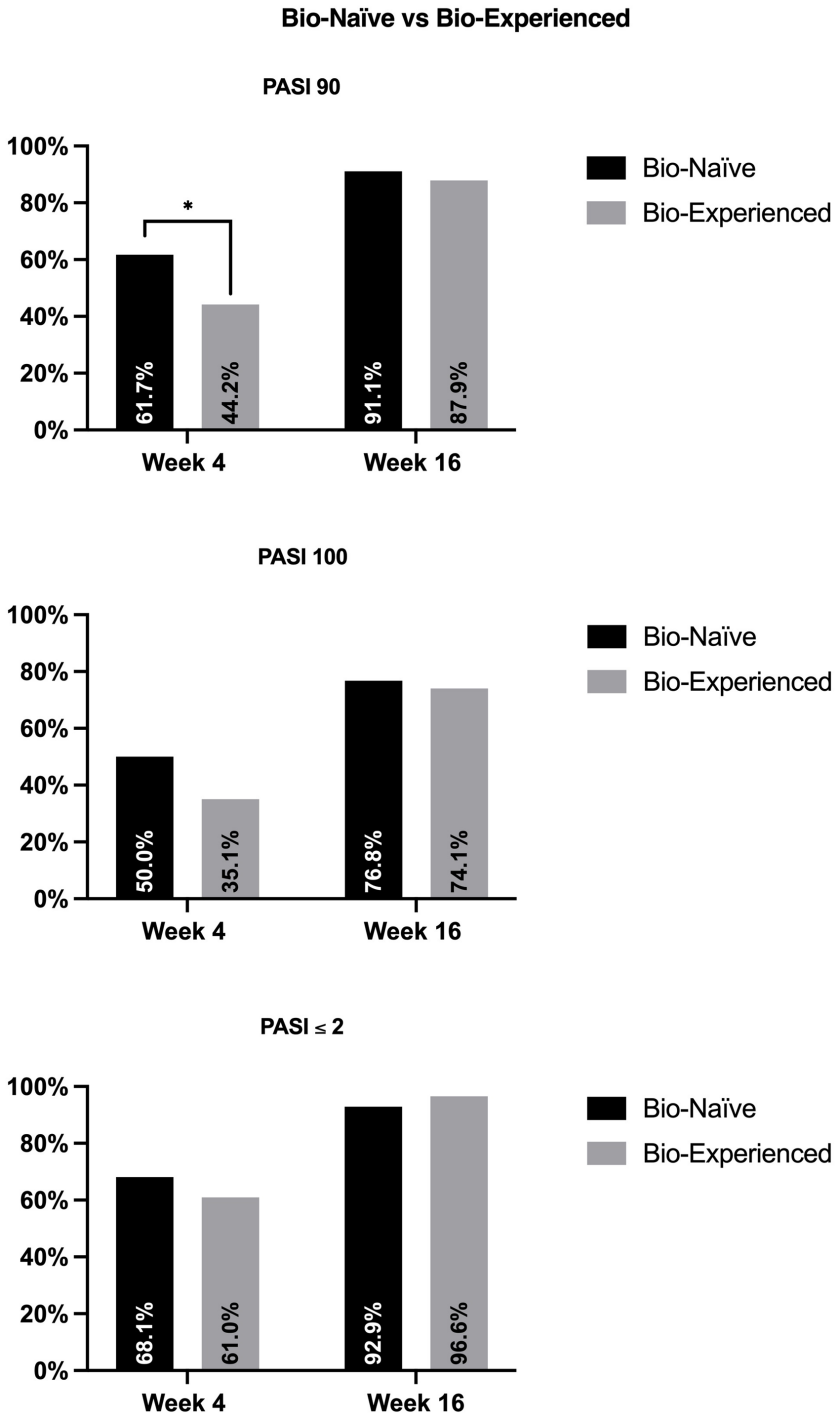
Effectiveness of bimekizumab according to previous exposure to biologics. PASI, psoriasis area and severity index. ^*^p-value <0.05.

Regarding the previous exposure to biologics (Figure 2), at week 4, bio-naïve patients were more likely to achieve PASI90 (61.7% versus 44.2%, *p* = 0.022) compared with the bio-experienced cohort. At the same time point, we did not observe significant differences in terms of PASI100 and PASI ≤2 (50% vs. 35.1%, *p* = 0.05; 68.1% vs. 61.0, *p* = 0.337). After 16 weeks of treatment, no significant differences were detected between the two groups regarding all effectiveness endpoints.

Regarding safety, only two patients (0.8%) discontinued the drug due to moderate eczematous reactions one and 2 weeks after the first dose of bimekizumab, respectively (Table 2). The most frequently reported AEs were oral candidiasis (10.1%) and upper respiratory tract infections (8.4%). There were no severe AEs or new cases of inflammatory bowel disease reported during the study. Additionally, none of the patients diagnosed with viral hepatitis or tuberculosis experienced reactivation.

**Table 2:** Safety profile of bimekizumab throughout the study period.

Event	No. (%)
Any AE	59 (24.9)
Severe AE	0
AE leading to discontinuation	2 (0.8)
Upper Respiratory Tract Infection	20 (8.4)
Oral Candidiasis	24 (10.1)
Nasopharyngitis	9 (3.8)
Diarrhea	2 (0.8)
Eczematous Reaction	2 (0.8)

AE, adverse event.

## 4. Discussion

Real-world experiences with bimekizumab are currently limited to case reports (7, 8). Our population at baseline had similar characteristics compared with phase-3 clinical trials (3–6). An exception was represented by the lower mean PASI at baseline in our experience because of the strict inclusion criteria of clinical trials. Interestingly, compared with those studies, our patients had a higher mean DLQI, highlighting a significant impact of the disease on their quality of life.

Compared with the randomized clinical trials, we observed similar or slightly higher rates of PASI90 and PASI100 at both time points. At week 4, PASI90 and PASI100 responses were higher in our population compared with the BE READY trial (53.8% vs. 45.3%; 43.3% vs. 18.9%). At week 16, our experience showed better results again in terms of complete skin clearance, compared with the be radiant (61.7%), be ready (68.2%), be vivid (59%) and be sure (60.8%) trials (3–6).

Regarding the impact of the treatment with bimekizumab on patients’ well-being, after 16 weeks, 86.8% of them reported that their psoriasis did not affect their quality of life (compared with 76 and 67% from the be ready and be vivid trials) (4, 5). This result is particularly meaningful, as our population had a high mean DLQI score at baseline (15.49).

In our experience, the safety profile of bimekizumab was comparable with data from clinical trials (11). No severe AEs were observed up to week 16. Mild oral candidiasis was the most frequently reported AE, but none of the patients discontinued the treatment because of this. No clinical or serological evidence of viral reactivation was observed in our patients with concomitant viral hepatitis, consistent with data reported for other IL-17 inhibitors (12).

In our study, the number of reported AEs was probably underestimated, as in clinical practice is uncommon for patients to report any mild AEs. This study has a few limitations, represented by its retrospective nature, the absence of a control group and the heterogeneity of clinical evaluations from different clinicians.

Our experience confirms the effectiveness of bimekizumab in the short term with comparable or better responses with respect to clinical trials. In this study, bio-naïve patients showed slightly better responses at week 4, but there were no significant differences after 16 weeks with the patients who were already treated with other biologics. In addition, no significant safety findings emerged throughout the study period.

## Data availability statement

The raw data supporting the conclusions of this article will be made available by the authors, without undue reservation.

## Ethics statement

Ethical review and approval was not required for the study on human participants in accordance with the local legislation and institutional requirements. The patients/participants provided their written informed consent to participate in this study.

## Author contributions

LG, AN, and LI contributed on all stage of this study, conception and design of the study, performed the statistical analysis, manuscript revision, read, and approved the submitted version. ACo and PM contributed to manuscript revision, read and approved the submitted version. AB, LB, PB, ACam, FC, AD, CD, PG, FL, AM, MMe, SM, AO, AP, GP, PQ, PS, and MV contributed to conception and design of the study, to data collection and approved the submitted version. DB, MB, GC, ACr, EC, CC, ACar, PD, FG, AG, EM, MMa, DO, LP, AR, and FR contributed to obtaining, analyzing and interpreting data, and approved the submitted version. All authors contributed to the article and approved the submitted version.

## Funding

This work was partially supported by “Ricerca Corrente” funding from Italian Ministry of Health to IRCCS Humanitas Research Hospital.

## Conflict of interest

LG has been a consultant for Almirall. PM has been a speaker for AbbVie, Lilly, Novartis, Janssen-Cilag, Celgene, Leopharma, and Almirall. AB has received honoraria for participation in advisory boards, meetings, or as speaker for AbbVie, Celgene, Janssen-Cilag, Eli Lilly, Novartis Pharma, Pfizer, Sanofi-Genzyme, and UCB Pharma. LB has received honoraria as a speaker or consultant for AbbVie, Janssen, Almirall Eli-Lilly, Leopharma, Novartis, Sanofi, Pfizer, and UCB Pharma. GC reports consulting fees or honorarium and payment for lectures from Lilly and Novartis. CD reports consulting fees or honorarium from Abbvie, Amgen, Novartis, Celgene, Sanofi, UCB Pharma, Janssen, and Lilly and payment for lectures from Abbvie, Lilly, Novartis, UCB Pharma, and Celgene. PG has been a consultant and/or speaker for Abbvie, Almirall, Amgen, Janssen, Leo-Pharma, Eli-Lilly, Novartis, Pierre Febre, Sandoz, Sanofi, and UCB. MB has acted as a speaker and consultant for AbbVie, Janssen, Amgen, Novartis, Eli Lilly, and UCB Pharma. CC has served as a board participant or speaker for Abbvie, Lilly, Janssen, Novartis, Celgene, Almirall, and Leopharma. PD has been a speaker for Novartis, Abbvie, Sanofi, UCB, Janssen, Lilly, and LeoPharma. FG acted as a speaker or consultant for Novartis, Abbvie, Eli Lilly, Celgene, LeoPharma, and Almirall. AG received grants or is an investigator for Biogen and Lilly; and is a consultant/advisory board/speaker for AbbVie, Almirall, Celgene, Janssen, Leo Pharma, Eli Lilly, Merck Sharpe Dohme, Novartis, Pfizer, Sandoz, and UCB. FL served on advisory boards and/or received honoraria for lectures from Abbvie, Janssen-Cilag, Novartis, Lilly, Sanofi. AM reports consultancy/advisory boards disease-relevant honoraria from AbbVie, Boehringer-Ingelheim, Novartis, Pfizer, Sanofi, and UCB. MMe acted as a speaker or consultant for Abbvie, Eli Lilly, Janssen, Leo-Pharma, UCB, and Novartis. AO acted as a speaker and consultant for Abbvie, Eli Lilly, Novartis, Celgene, Sanofi, Galderma, Leo Pharma, and Pierre Fabre. MV served as a speaker or advisory board member for Abbvie, Almirall, Amgen, Eli-Lilly, Galderma, Leo Pharma, Novartis, Pierre Fabre, and UCB Pharma. ACo has served as an advisory board member, consultant and has received fees and speaker’s honoraria or has participated in clinical trials for Abbvie, Almirall, Biogen, LEO Pharma, Lilly, Janssen, Novartis, Pfizer, Sanofi Genzyme, and UCB-Pharma. AN has served on advisory boards, received honoraria for lectures and research grants from Almirall, Abbvie, Leo Pharma, Celgene, Eli Lilly, Janssen, Novartis, Sanofi-Genzyme, Amgen, and Boehringer Ingelheim.

The remaining authors declare that the research was conducted in the absence of any commercial or financial relationships that could be construed as a potential conflict of interest.

## Publisher’s note

All claims expressed in this article are solely those of the authors and do not necessarily represent those of their affiliated organizations, or those of the publisher, the editors and the reviewers. Any product that may be evaluated in this article, or claim that may be made by its manufacturer, is not guaranteed or endorsed by the publisher.

## References

[1] Armstrong AW Read C. Pathophysiology, clinical presentation, and treatment of psoriasis: a review. JAMA. (2020) 323:1945–60. doi: 10.1001/jama.2020.4006 32427307

[2] Kolbinger F Loesche C Valentin MA Jiang X Cheng Y Jarvis P. β-defensin 2 is a responsive biomarker of IL-17A-driven skin pathology in patients with psoriasis. J Allergy Clin Immunol. (2017) 139:923–932.e8. doi: 10.1016/j.jaci.2016.06.038, PMID: 27502297

[3] Warren RB Blauvelt A Bagel J Papp KA Yamauchi P Armstrong A. Bimekizumab versus adalimumab in plaque psoriasis. N Engl J Med. (2021) 385:130–41. doi: 10.1056/NEJMoa2102388, PMID: 33891379

[4] Gordon KB Foley P Krueger JG Pinter A Reich K Vender R. Bimekizumab efficacy and safety in moderate to severe plaque psoriasis (be ready): a multicentre, double-blind, placebo-controlled, randomised withdrawal phase 3 trial. Lancet. (2021) 397:475–86. doi: 10.1016/S0140-6736(21)00126-4 33549192

[5] Reich K Papp KA Blauvelt A Langley RG Armstrong A Warren RB. Bimekizumab versus ustekinumab for the treatment of moderate to severe plaque psoriasis (be vivid): efficacy and safety from a 52-week, multicentre, double-blind, active comparator and placebo controlled phase 3 trial. Lancet. (2021) 397:487–98. doi: 10.1016/S0140-6736(21)00125-2, PMID: 33549193

[6] Reich K Warren RB Lebwohl M Gooderham M Strober B Langley RG. Bimekizumab versus secukinumab in plaque psoriasis. N Engl J Med. (2021) 385:142–52. doi: 10.1056/NEJMoa2102383, PMID: 33891380

[7] Valenti M Gargiulo L Ibba L Pavia G Narcisi A Costanzo A. Sub-erythrodermic psoriasis successfully treated with bimekizumab: a case report. Dermatol Ther. (2022) 35:e15952. doi: 10.1111/dth.15952, PMID: 36269298 PMC10078003

[8] Megna M Battista T Potestio L Ruggiero A Ventura V Fabbrocini G. A case of erythrodermic psoriasis rapidly and successfully treated with bimekizumab. J Cosmet Dermatol. (2023) 22:1146–8. doi: 10.1111/jocd.15543, PMID: 36448190

[9] Gisondi P Fargnoli MC Amerio P Argenziano G Bardazzi F Bianchi L. Italian adaptation of EuroGuiDerm guideline on the systemic treatment of chronic plaque psoriasis. Ital J Dermatol Venerol. (2022) 157:1–78. doi: 10.23736/S2784-8671.21.07132-2, PMID: 35262308

[10] European Medicines Agency. Bimzelx (bimekizumab): summary of product characteristics. (2023). Available at: https://www.ema.europa.eu/en/documents/product-information/bimzelx-epar-product-information_it.pdf. (Accessed April 28, 2023)

[11] Gordon KB Langley RG Warren RB Okubo Y Stein Gold L Merola JF. Bimekizumab safety in patients with moderate to severe plaque psoriasis: pooled results from phase 2 and phase 3 randomized clinical trials. JAMA Dermatol. (2022) 158:735–44. doi: 10.1001/jamadermatol.2022.1185, PMID: 35544084 PMC9096693

[12] Gargiulo L Pavia G Valenti M Lleo de Nalda A Perugini C Costanzo A. Safety of biologic therapies in patients with moderate-to-severe plaque psoriasis and concomitant viral hepatitis: a monocentric retrospective study. Dermatol Ther. (2022) 12:1263–70. doi: 10.1007/s13555-022-00726-w, PMID: 35460018 PMC9110615

